# Metastatic Renal Cell Carcinoma to Pancreas: Case Series and Review of the Literature

**DOI:** 10.3390/diagnostics13081368

**Published:** 2023-04-07

**Authors:** Daniel Vasile Balaban, Laura Coman, Flavius Stefan Marin, Marina Balaban, Daniela Tabacelia, Florina Vasilescu, Raluca Simona Costache, Mariana Jinga

**Affiliations:** 1 Internal Medicine and Gastroenterology, Carol Davila University of Medicine and Pharmacy, 050474 Bucharest, Romania; 2 Gastroenterology Department, Central Military Emergency University Hospital, 010825 Bucharest, Romania; 3 Gastroenterology Department, Saint Mary’s Clinical Hospital, 011172 Bucharest, Romania; 4 Pathology Department, Central Military Emergency University Hospital, 010825 Bucharest, Romania

**Keywords:** pancreas, metastasis, renal cell carcinoma, hypervascular

## Abstract

Metastasis to the pancreas represents a small proportion of all pancreatic malignancies. Among primary tumors that metastasize to the pancreas, renal cell carcinoma (RCC) is one of the most common causes of metastatic pancreatic lesions. We herein report a case series of three patients with pancreatic metastasis from RCC. The first is a 54-year-old male with a history of left nephrectomy for RCC, in whom an isthmic pancreatic mass suggestive of a neuroendocrine lesion was found during oncological follow-up. Endoscopic ultrasound (EUS)-guided fine needle biopsy (FNB) identified pancreatic metastasis of RCC and the patient was referred for surgery. The second case is a 61-year-old male, hypertensive, diabetic, with left nephrectomy for RCC six years previously, who complained of weight loss and was found with a hyperenhancing mass in the head of the pancreas and a lesion with a similar pattern in the gallbladder. EUS-FNB from the pancreas proved to be a metastatic pancreatic lesion. Cholecystectomy and treatment with tyrosine kinase inhibitors were recommended. The third case is a 68-year-old dialysis patient referred for evaluation of a pancreatic mass, also confirmed by EUS-FNB, who was started on sunitinib treatment. We report a literature summary on epidemiology and clinical features, diagnosis and differential diagnosis and treatment and outcomes in pancreatic metastasis of RCC.

## 1. Introduction

Decades ago, all pancreatic masses were thought to be carcinomas; however, with the improvements in diagnostic techniques, it is now recognized that up to one in four solid pancreatic lesions are not ductal adenocarcinoma, and differential diagnoses include inflammatory masses, lymphoma, neuroendocrine tumors, metastases and other rare lesions. Moreover, with advances in tissue sampling, we can further refine treatment and prognosis by accurately diagnosing various histological subtypes of pancreatic neoplasia [1].

The pancreas is a rare site for metastasis compared with other parenchymal abdominal organs. Pancreatic metastases (PM) are rarely encountered in clinical practice and account for only about 2–5% of all pancreatic malignancies [2,3]. The most common causes of PM include renal cell carcinoma (RCC), melanoma and breast, ovarian and colon cancers [4].

PM from RCC is among the most frequent site of metastasis for this type of neoplasia, and about half of all PM are caused by RCC [5]. In contrast to the dismal prognosis for metastatic diseases in general, metastatic RCC to pancreas has a particularly favorable outcome.

We herein present a case series of three patients with pancreatic metastasis of renal cell carcinoma and review the literature, focusing on epidemiology, diagnosis and treatment options.

## 2. Case Presentations

### 2.1. Case 1

**Figure 1:**
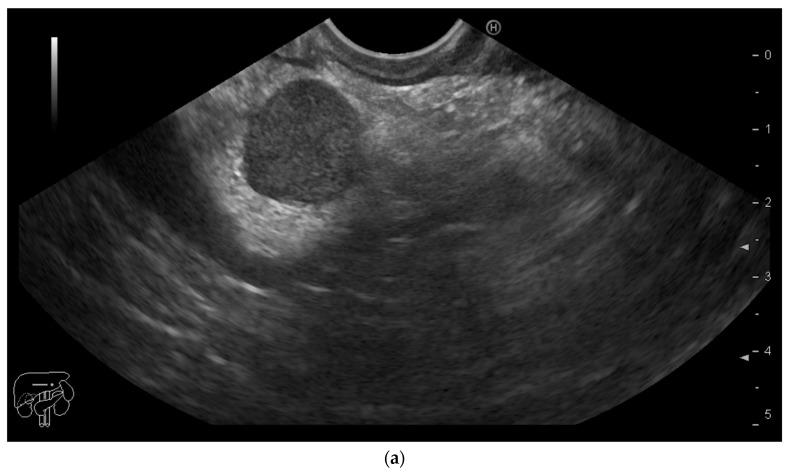

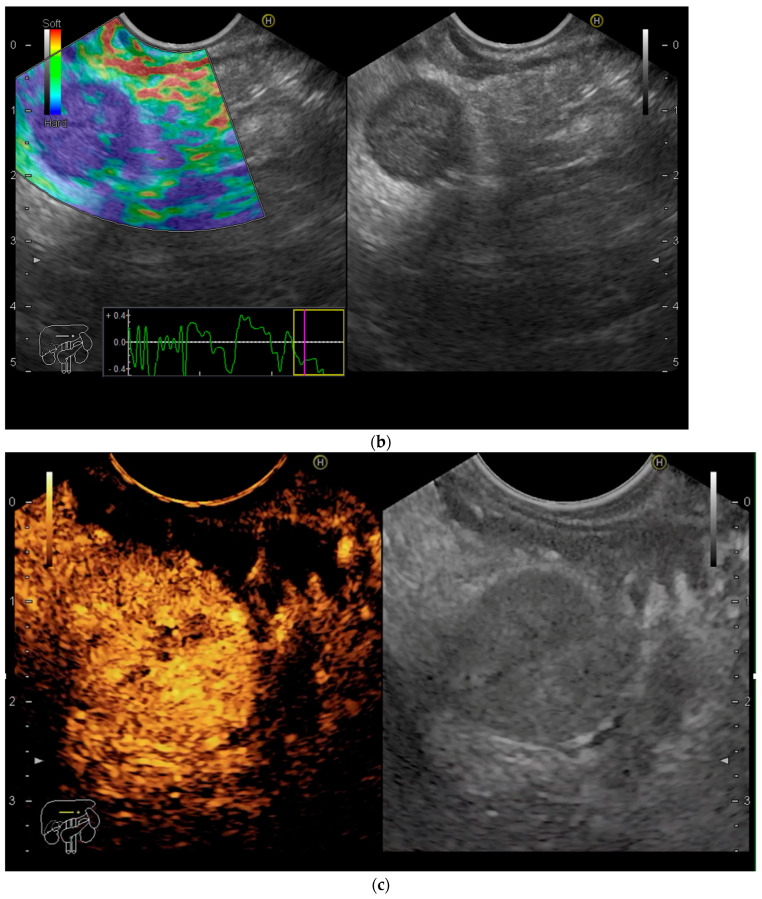

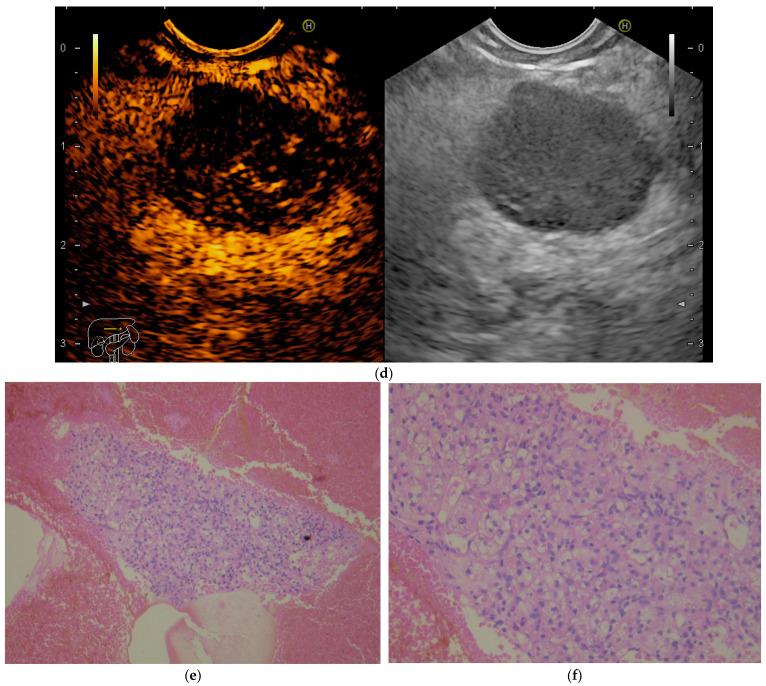
(**a**) Endoscopic ultrasound using a linear probe: hypoechoic mass in the isthmus of the pancreas. (**b**) Real-time elastography showing a hard homogeneous pattern in the lesion. (**c**) Contrast-enhanced EUS showing a hyperenhancing pattern of the lesion in the arterial phase. (**d**) Contrast-enhanced EUS image showing washout of the lesion in the late phase. (**e**,**f**)—Hematoxylin-eosin stain, 100× and 200× magnitude, showing RCC tumor proliferation.

A 54-year-old male ex-smoker was referred by the oncologist for evaluation of a pancreatic mass. Personal medical history was remarkable for left nephrectomy and adrenalectomy 6 months before, for RCC. Follow-up imaging—computed tomography and magnetic resonance imaging—revealed a 16/18 mm pancreatic mass suggestive of a neuroendocrine tumor. He had no digestive complaints. Physical examination was unremarkable, except for a post-operative left flank scar. Laboratory work-up showed mild normocytic anemia. Tumor markers (CEA, CA 19-9, CA 125), serum chromogranin, serotonin and urinary 5-hydroxyindoleacetic acid were within normal limits. We performed endoscopic ultrasound (EUS) to further characterize the pancreatic mass and obtain a histopathologic diagnosis. EUS showed a well-delineated, round hypoechoic lesion in the pancreatic isthmus (Figure 1a), with a hard homogeneous pattern on elastography (Figure 1b). Upon administration of contrast media (SonoVue), rapid uptake of contrast was seen in the arterial phase, with washout in the late phase (Figure 1c,d). A fine-needle biopsy (FNB) was performed using a 20 G ProCore needle, and the histopathology report confirmed the diagnosis of metastatic RCC in the pancreas (Figure 1e,f). Following the oncology board’s decision, the patient was referred for surgery. No recurrence of metastatic lesions was found on imaging at the one-year follow-up.

### 2.2. Case 2

**Figure 2:**
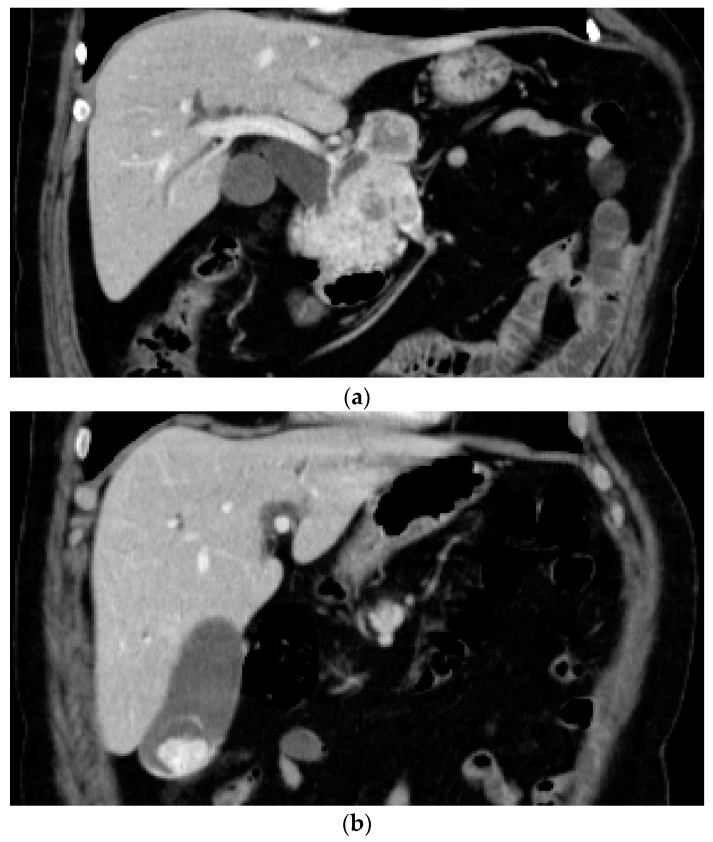
(**a**) Contrast-enhanced computed-tomography scan showing hyperenhancing mass in the head of the pancreas, with consecutive common bile duct dilation. (**b**) Contrast-enhanced computed-tomography scan showing hyperenhancing mass in the fundus of the gallbladder.

A 61-year-old male presented to the gastroenterology department with involuntary weight loss and upper abdominal pain. The patient was suffering from hypertension and type 2 diabetes mellitus, for which he was taking calcium channel blockers, an angiotensin receptor blocker and insulin. He had undergone left nephrectomy for RCC 6 years previously. Physical examination revealed an overweight patient with a postoperative abdominal scar and orthostatic hypotension. Laboratory examinations revealed mild normocytic anemia, elevated HbA1c and a decreased glomerular filtration rate. A CT-scan revealed a 6/5 cm hyperenhancing mass in the head of the pancreas, with consecutive dilatation of the common bile duct, and a 28/18 mm lesion with similar enhancing pattern in the gallbladder (Figure 2a,b), suggestive of metastasis, considering the RCC history. EUS-FNB of the pancreatic mass confirmed the diagnosis of RCC metastasis. Cholecystectomy and treatment with tyrosine kinase inhibitors were recommended.

### 2.3. Case 3

**Figure 3:**
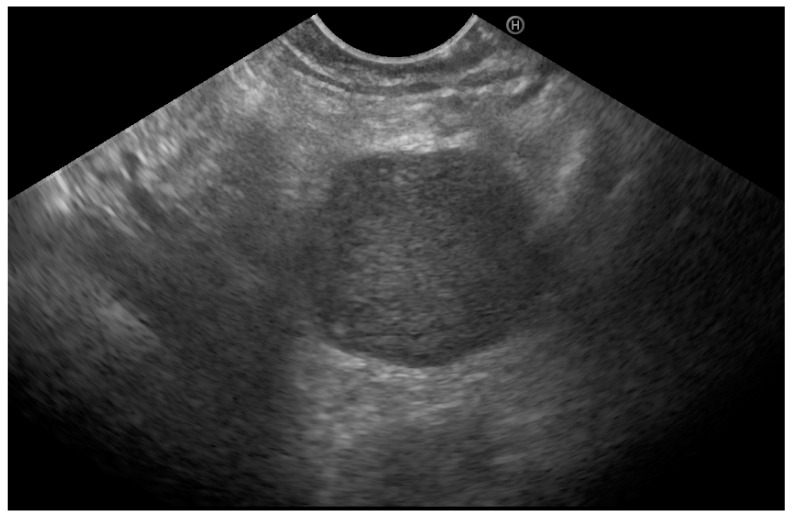
Endoscopic ultrasound using a linear probe positioned in the duodenum, showing a hypoechoic round mass in the head of the pancreas.

A 68-year-old dialysis patient presented for further evaluation of a pancreatic mass detected on ultrasound and confirmed by CT scan. He was complaining of postprandial bloating but had overall good performance status. Three years before the presentation, the patient had undergone right nephrectomy for Grawitz tumor and was currently undergoing dialysis. Laboratory workup showed considerably decreased glomerular filtration rate and mild normocytic anemia, with no other specific findings. Tumor markers (CEA, CA 19-9, CA 125) were within normal limits. EUS revealed a 25 mm cephalopancreatic mass (Figure 3) with a hard homogeneous pattern on elastography and a hyperenhancing pattern on contrast administration (SonoVue). Transduodenal FNB with a 20G needle confirmed the mass to be an RCC metastatic lesion. The patient was started on sunitinib treatment. The patient did not show disease progression at the 2-year follow-up.

## 3. Search Strategy

Considering the heterogeneous clinical and imaging features revealed by our case series, we aimed to summarize currently available evidence on the epidemiology, diagnosis and outcomes of PM from RCC in order to delineate a pathway for the diagnostic approach for a patient with a history of renal malignancy in whom a pancreatic mass is detected. For this purpose, we searched PubMed in December 2022 for all publications on the association between PM and RCC using a combination of the search terms “renal cell carcinoma” and “pancreas metastasis”. The search yielded 358 results, which we filtered by title and abstract for relevance on the topic. Papers selected for full-text analysis were further screened for potentially relevant additional references and cited articles that might have been missed in the initial search. Records selected for the literature review were grouped according to the topic covered: epidemiology, clinical features, diagnostic instruments and outcome.

## 4. Discussion

RCC is characterized by a broad metastatic spectrum, but among metastatic involvement of organs, the pancreas seems to be a frequent site [6]. The pancreatic tropism of metastatic RCC is defined by a particularly favorable outcome and seems to be associated with the indolent behavior of the primary tumor, compared with RCC metastasizing to other sites [6,7]. This has enabled authors to conclude that the presence of PM in the setting of RCC is an indicator of a disease course with good prognosis [8,9]. While the presence of a pancreatic mass is usually associated with dismal outcomes of pancreatic ductal adenocarcinoma (PDAC), PM from RCC are usually highly responsive to therapy and are associated with long-term survival, in contrast to the aggressive tumor biology and chemoresistance of pancreatic cancer. Additionally, in contrast to PM associated with other neoplasia, which is an expression of systemic disseminated disease, metastatic RCC to the pancreas is a more localized determination of the disease and is frequently amenable to surgery [2].

### 4.1. Mechanism of Metastasis

The pathway to metastasis occurrence in the pancreas from RCC is mainly hematogenous. Metastatic lesions can be either solitary or multiple. The mechanism of isolated PM in RCC involves a particular “seed and soil” pathway that explains the selective development of metastases in the pancreas by embolized cells from the primary tumor. This specific metastatic mechanism creates a frame for uniform distribution of metastatic lesions across the pancreas, independent of the RCC site, and similar outcomes in singular versus multiple, synchronous versus metachronous isolated PM [10,11,12,13,14].

### 4.2. Epidemiology and Clinical Features

PM from RCC can be synchronous or metachronous. Because of the potential for late occurrence of metastasis, long-term follow-up is recommended in patients with a history of RCC [15]. PM can also present as single or multiple masses, separately or associated with other types of pancreatic lesions [16].

PM are often asymptomatic, with a slow-growing pattern and indolent tumor biology [17]. With regard to the temporal relationship between RCC and PM, renal neoplasia can be diagnosed before or after detection of the pancreatic lesions [18,19]. Metachronous metastasis can occur long after primary tumor manifestation (up to 3 decades later) [20,21]. In our case series, the time to PM occurrence following nephrectomy varied from 6 months to 6 years.

While diagnosis usually starts with detecting a pancreatic mass during patient follow-up or identification of symptoms associated with cancer cachexia, the initial presentation can be an emergency such as gastrointestinal bleeding, pancreatitis or jaundice [22,23,24,25,26].

As for PDAC, diabetes mellitus (DM)—and particularly new-onset DM—can be an indicator of possible pancreatic involvement in the evolution of a patient with a history of RCC [27,28,29]. Interestingly, among host-related factors, fatty pancreas has been reported as a risk factor for late pancreatic metastases from RCC [30].

The site and frequency of metastatic involvement depends on the histologic subtype of RCC—clear cell (ccRCC), papillary (pRCC), spindle cell type (sRCC) and chromophobe (chrRCC). PM are most frequently seen in ccRCC [31,32,33]. PM from RCC have also been reported in ectopic pancreatic tissue, such as in the case reported by Yano et al. [34]. Moreover, as reported in one of our cases, digestive involvement of RCC metastasis includes gastrointestinal tract (stomach, duodenum, colon), ampulla of Vater and gallbladder in addition to the pancreas. Metastases can occur simultaneously in GI sites, as in our case, or at different timepoints during follow-up [20,35,36,37,38,39,40,41,42,43,44,45,46,47,48,49,50,51,52].

### 4.3. Diagnosis

PM can be detected via abdominal imaging, starting from initial evaluation with conventional ultrasound and confirmation with cross-sectional techniques such as computed tomography (CT) or magnetic resonance imaging (MRI).

On CT scans, PM characteristics resemble those of primary RCC, i.e., well delineated and hyperenhancing compared with the surrounding parenchyma [29]. Similar to the pattern of the primary tumor, there is a rapid uptake of contrast and rapid washout on contrast-enhanced CT in a “fast-in and fast-out” pattern [23]. Besides the mass, another imaging feature that can be seen is that of tumoral thrombus in the MPD, as described by Momose et al. [53]. While the homogeneous intense hyperenhancement is typical for small PM, only rim enhancement can be seen in larger lesions [54].

The use of fluorodeoxyglucose (FDG)-positron emission tomography (PET) in the assessment of metastatic RCC has not been extensively studied [3]. While some studies have reported modest sensitivity—in the range of 63.6%—in detecting metastasis from RCC [55], and particularly small metastatic lesions, others have concluded that FDG-PET is an efficient instrument for proving disease recurrence at metastatic sites [56].

**Figure 4:**
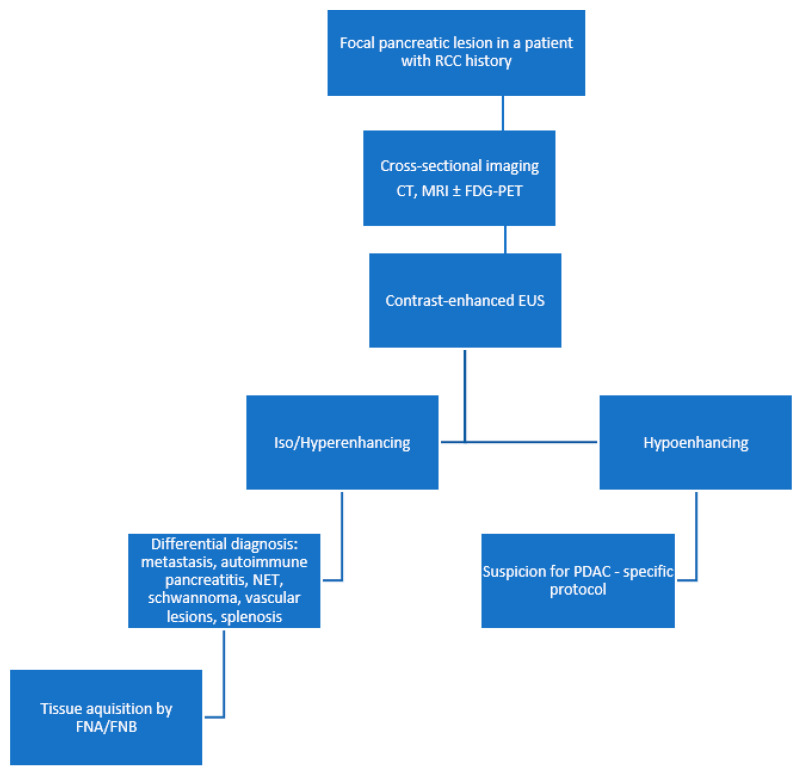
Diagnostic algorithm for a patient with solid pancreatic lesion and a history of RCC.

Endoscopic ultrasound (EUS) allows further characterization and tissue sampling of a suspicious pancreatic mass by means of fine-needle aspiration (FNA) or fine-needle biopsy (FNB). PM appear as hypoechoic, well delineated solid tumors on EUS [57]. Additional imaging techniques such as contrast-enhancement (CE-EUS) and elastography (EUS-E) can be useful for differential diagnosis of a solid pancreatic lesion. PM from RCC are hyperenhanced, as seen in our cases, but PM from other primary tumors, such as colon or breast cancers, can also be iso- or hypoenhanced [58]. The hyperenhancing patterns of PM easily differentiate them from PDAC, which is hypoenhanced. Based on the EUS-E, the PM reported in our cases had a stiff appearance, similar to PDAC and NET. Quantitative elastography and the validated cut-offs for malignancy—strain ratio > 10 and strain histogram < 50—have identified PM as hard lesions [59]. In a multicenter study looking at small pancreatic lesions, including 8% PM (of which more than half were from RCC), 59% of the cases were reported as stiff and 41% were reported as soft [60]. The same study reported atypical behavior for small PDAC, with two lesions being soft and one being isoenhanced. Considering the patterns of PM reported in the literature, there is a consistent hyperenhancement appearance on CE-EUS, but variable stiffness on EUS-E. Thus, based on the contrast-enhanced imaging findings of a solid pancreatic lesion detected in a patient with RCC history, an algorithm is foreseen—Figure 4,

Tissue acquisition from PM using both FNA and FNB needles has been reported [61,62]. EUS-FNA has high diagnostic accuracy, even in cytology and using cell-blocks [63,64]; however, results are even better when biopsy needles, which take histological samples, are used. In a study of 672 solid pancreatic masses, among which 53 were PM, EUS-FNA had 84.9% sensitivity and 100% specificity for diagnosing PM [65]. Specific puncturing techniques have been recommended to improve diagnosis, including short aspiration with low negative vacuum pressure, in the French series reported by Béchade et al. [66].

A table summarizing the clinical and diagnostic features of PM from RCC, with data pooled from selected papers identified during the literature search, is available as Supplementary Table S1. Overall, there is significant heterogeneity in the diagnostic features of RCC-PM, particularly first-line imaging studies performed either as routine follow-ups or directed by clinical manifestations. Thus, due to the large variability in clinical presentations, some patients with jaundice were first evaluated using abdominal ultrasound, others with ampullary lesions or bile duct obstruction and cholangitis underwent endoscopic retrograde cholangiopancreatography (ERCP), while patients presenting with upper gastrointestinal bleeding were first evaluated endoscopically. A review of diagnostic routes for patients with PM showed that many publications lack information on pre-operative histopathologic confirmation of the lesions.

### 4.4. Differential Diagnosis

The most common differential diagnosis on imaging is pancreatic neuroendocrine tumor (pNET), since both are hypervascular [67,68]. Other hypervascular pancreatic lesions include neurogenic tumors such as schwannoma, vascular lesions or intrapancreatic accessory spleen [69]. Additionally, PM can occur in patients with primary tumors other than RCC for example melanoma and breast or ovarian neoplasia. In this setting, a thorough anamnesis to reveal the history of nephrectomy for RCC is of paramount importance. Another confusing factor is that PM can also show uptake on DOTATATE PET/CT scan, a technique used for NET [70]. There are some useful imaging indicators which can be used to differentiate pNET from RCC-PM, such as the relative percentage washout (RPW) value [71].

Differential diagnosis can be even more challenging, as tumor-to-tumor metastasis has been reported, such as the case of an RCC metastasizing to a pancreatic endocrine neoplasm or microcystic serous cystadenoma [72,73,74].

A major stake in the differential diagnosis is with PDAC. While differential diagnosis with PDAC seems straightforward—with one being hypovascular and the other being hypervascular—synchronous PM from RCC and PDAC have been reported in the literature [75]. Synchronous RCC and PDAC have also been described in case reports [76]. In cases where differential diagnosis is challenging, IHC markers such as PAX2 and mesothelin may be useful for distinguishing PDAC from PM from RCC [77,78].

Finally, a very rare encounter is that of primary clear cell carcinoma of the pancreas [79]. Differential diagnosis with IPMN has been reported in some cases also [80].

### 4.5. Treatment and Outcome

PM-RCC treatment includes surgery, ablative and medical therapy. Compared with other sites of metastasis, PM from RCC have better prognosis [81]. Additionally, synchronous metastasis can be seen in other sites and the pancreas, and can be addressed during the same surgical session [82,83,84].

Surgery for PM is safe, has a low rate of complications, and is considered the main therapy for oligometastatic disease [15,85]. All types of surgical resection—pancreatoduodenectomy, central pancreatectomy, distal pancreatectomy, total pancreatectomy and atypical resections (enucleation or enucleo-resection)—have been described in PM from RCC, with good outcomes in several surgical series [86,87,88,89,90,91,92,93,94,95,96,97]. Surgery is indicated even in the case of multifocal pancreatic involvement [98]. Due to uncommon lymph node involvement, extensive node dissection is not usually required [99]. Despite the favorable outcome associated with PM surgery, we should keep in mind the high morbidity of pancreatic surgery, which is reported to be up to 34%. While adequate resection margins should be achieved, parenchymal sparing surgery is warranted in order to decrease postoperative morbidity in these patients [100,101].

Other studies have reported that surgical resection of PM has no significant benefit on survival, in the setting of tyrosine kinase inhibitors (TKI) [102,103].

While long progression-free periods can be seen after pancreatic metastasectomy, disease recurrence can occur and long-term follow-up should therefore be conducted in these patients [81,104]. Repeat resection in the remnant pancreas in case of additional metachronous lesions after initial PM surgery has been also reported [105]. Repeat surgery can also be indicated for recurrence in the periampullary region following previous metastasis in distal pancreas [106].

Besides surgery, successful ablative techniques such as radiofrequency ablation (RFA) have also been reported for PM of RCC [107]. In recent years, EUS-guided RFA has emerged as a promising technique for treating various pancreatic lesions, including pNET, pancreatic cystic neoplasms and PDAC [108,109]. The systematic review by Dhaliwal et al. [110] looking at technical and clinical success rates of EUS-RFA found that of 134 patients treated by RFA, only 4 (3%) had PM, 3 of them from RCC. In one study reporting on a case of PM from RCC [111], the indication for EUS-RFA was set for a patient with chronic renal failure who was unsuitable for surgery or chemotherapy. The prospective series by Chanez et al. [112] describing 26 EUS-RFA on 12 patients reported 40% complete response at 12 months, with 2 severe complications (a duodenal abscess and a liver abscess). The efficacy of the ablation can be seen readily during contrast-enhanced follow-up imaging, particularly in the case of RCC-PM, which is hypervascular, and depicts a non-enhancing area instead of the tumor. In case of incomplete necrosis and evidence of contrast-enhancing remnant tissue, a second RFA session can be performed.

A combination of targeted therapy and immunotherapy can be used where disease progression occurs after surgical resection of the metastasis [113]. PM are highly responsive to tyrosine kinase inhibitors, and complete radiologic responses have been reported in some cases [114,115]. Response to targeted therapy is, however, organ-specific; while PM have good responses, liver metastases have poor responses [116]. Same site-specific responsivity has been reported for nivolumab in metastatic RCC, with 33% overall response rate for the pancreas [117].

## 5. Conclusions

The pancreas is a predilect site of metastasis from RCC and can present either as a single or multiple lesion. PM can be asymptomatic and can be detected during follow-up imaging of an oncologic patient or can manifest as a mass lesion in the upper abdomen. As PM can occur long after initial diagnosis of the primary tumor, lifelong follow-up is therefore recommended for RCC patients. Upon detection of a pancreatic mass, high-resolution imaging and tissue acquisition should be conducted in order to accurately diagnose PM from RCC. Its hyperenhancing pattern discriminates it from PDAC, but differential diagnosis of other hypervascular lesions, particularly neuroendocrine tumors, is required. Despite being very heterogenous lesions, PM have good overall prognosis.

## Data Availability

The dataset is available from the corresponding author.

## Supplementary Materials

The following supporting information can be downloaded at: https://www.mdpi.com/article/10.3390/diagnostics13081368/s1, Table S1: Summary of clinical and diagnostic features of pancreatic metastatic lesions.

## References

[1] Bazzichetto C., Luchini C., Conciatori F., Vaccaro V., Di Cello I., Mattiolo P., Falcone I., Ferretti G., Scarpa A., Cognetti F. (2020). Morphologic and Molecular Landscape of Pancreatic Cancer Variants as the Basis of New Therapeutic Strategies for Precision Oncology. Int. J. Mol. Sci..

[2] Cheng S.K.H., Chuah K.L. (2016). Metastatic Renal Cell Carcinoma to the Pancreas: A Review. Arch. Pathol. Lab. Med..

[3] Ballarin R., Spaggiari M., Cautero N., De Ruvo N., Montalti R., Longo C., Pecchi A., Giacobazzi P., De Marco G., D’Amico G. (2011). Pancreatic metastases from renal cell carcinoma: The state of the art. World J. Gastroenterol..

[4] Cortez N., Berzosa M., Mahfouz M., Dvir K., Galarza Fortuna G.M., Ben-David K. (2020). Diagnosis and Treatment of Metastatic Disease to the Pancreas. J. Laparoendosc. Adv. Surg. Technol. A.

[5] Li J., Liu Y., Deng T., Yang S., Chen J., Wu W., He Y. (2019). Pancreatic metastasis of renal clear cell carcinoma: A case report and literature review. Dig. Med. Res..

[6] Singla N., Xie Z., Zhang Z., Gao M., Yousuf Q., Onabolu O., McKenzie T., Tcheuyap V.T., Ma Y., Choi J. (2020). Pancreatic tropism of metastatic renal cell carcinoma. JCI Insight.

[7] Cignoli D., Fallara G., Aleotti F., Larcher A., Rosiello G., Rowe I., Basile G., Colandrea G., Martini A., De Cobelli F. (2022). Pancreatic metastases after surgery for renal cell carcinoma: Survival and pathways of progression. World J. Urol..

[8] Chrom P., Stec R., Bodnar L., Szczylik C. (2018). Prognostic Significance of Pancreatic Metastases from Renal Cell Carcinoma in Patients Treated with Tyrosine Kinase Inhibitors. Anticancer Res..

[9] Grassi P., Verzoni E., Mariani L., De Braud F., Coppa J., Mazzaferro V., Procopio G. (2013). Prognostic role of pancreatic metastases from renal cell carcinoma: Results from an Italian center. Clin. Genitourin. Cancer.

[10] Sellner F. (2020). Isolated Pancreatic Metastases of Renal Cell Carcinoma-A Paradigm of a Seed and Soil Mechanism: A Literature Analysis of 1034 Observations. Front. Oncol..

[11] Sellner F. (2018). Isolated pancreatic metastases from renal cell carcinoma: An outcome of a special metastatic pathway or of specific tumor cell selection?. Clin. Exp. Metastasis.

[12] Sellner F., Thalhammer S., Klimpfinger M. (2021). Tumour Evolution and Seed and Soil Mechanism in Pancreatic Metastases of Renal Cell Carcinoma. Cancers.

[13] Sellner F., Thalhammer S., Klimpfinger M. (2022). Isolated Pancreatic Metastases of Renal Cell Cancer: Genetics and Epigenetics of an Unusual Tumour Entity. Cancers.

[14] Sellner F. (2019). Observations on Solitary Versus Multiple Isolated Pancreatic Metastases of Renal Cell Carcinoma: Another Indication of a Seed and Soil Mechanism?. Cancers.

[15] Wente M.N., Kleeff J., Esposito I., Hartel M., Müller M.W., Fröhlich B.E., Büchler M.W., Friess H. (2005). Renal cancer cell metastasis into the pancreas: A single-center experience and overview of the literature. Pancreas.

[16] Benhaim R., Oussoultzoglou E., Saeedi Y., Mouracade P., Bachellier P., Lang H. (2015). Pancreatic metastasis from clear cell renal cell carcinoma: Outcome of an aggressive approach. Urology.

[17] Moletta L., Milanetto A.C., Vincenzi V., Alaggio R., Pedrazzoli S., Pasquali C. (2014). Pancreatic secondary lesions from renal cell carcinoma. World J. Surg..

[18] Thompson L.D., Heffess C.S. (2000). Renal cell carcinoma to the pancreas in surgical pathology material. Cancer.

[19] Gilbert C.M., Monaco S.E., Cooper S.T., Khalbuss W.E. (2011). Endoscopic ultrasound-guided fine-needle aspiration of metastases to the pancreas: A study of 25 cases. CytoJournal.

[20] Abdul-Ghafar J., Ud Din N., Saadaat R., Ahmad Z. (2021). Metastatic renal cell carcinoma to pancreas and gastrointestinal tract: A clinicopathological study of 3 cases and review of literature. BMC Urol..

[21] Nakamura H., Tanaka S., Miyanishi K., Kawano Y., Osuga T., Ishikawa K., Yoshida M., Ohnuma H., Murase K., Takada K. (2021). A case of hypervascular tumors in the liver and pancreas: Synchronous hepatocellular carcinoma and pancreatic metastasis from renal cell carcinoma 36 years after nephrectomy. Clin. Case Rep..

[22] Matsui S., Ono H., Asano D., Ishikawa Y., Ueda H., Akahoshi K., Ogawa K., Kudo A., Tanaka S., Tanabe M. (2021). Pancreatic metastasis from renal cell carcinoma presenting as gastrointestinal hemorrhage: A case report. J. Surg. Case Rep..

[23] Yamawaki M., Takano Y., Noda J., Azami T., Kobayashi T., Niiya F., Maruoka N., Yamagami T., Nagahama M. (2022). A case of hemobilia caused by pancreatic metastasis of renal cell carcinoma treated with a covered metallic stent. Clin. J. Gastroenterol..

[24] Gajendra S., Sachdev R., Mohapatra I., Goel R., Goel S. (2015). Metastatic Renal Cell Carcinoma: An Unusual Cause of Bleeding Pancreatic Mass. J. Clin. Diagn. Res. JCDR.

[25] Karakatsanis A., Vezakis A., Fragulidis G., Staikou C., Carvounis E.E., Polydorou A. (2013). Obstructive jaundice due to ampullary metastasis of renal cell carcinoma. World J. Surg. Oncol..

[26] Schauer M., Vogelsang H., Siewert J.R. (2008). Pancreatic resection for metastatic renal cell carcinoma: A single center experience and review of the literature. Anticancer Res..

[27] Jelleli N., Loghmari A., Belkacem O., Tlili G., Jellali B., Chouaya S., Bouassida K., Hmida W., Jaidane M., Hmissa S. (2022). Renal cell carcinoma with atypical metastases sites revealed by diabetes mellitus: A case report. Ann. Med. Surg..

[28] Mellenthin C., Balaban V.D., Dugic A., Cullati S. (2022). Risk Factors for Pancreatic Cancer in Patients with New-Onset Diabetes: A Systematic Review and Meta-Analysis. Cancers.

[29] Ghavamian R., Klein K.A., Stephens D.H., Welch T.J., LeRoy A.J., Richardson R.L., Burch P.A., Zincke H. (2000). Renal cell carcinoma metastatic to the pancreas: Clinical and radiological features. Mayo Clin. Proc..

[30] Fahlbusch T., Luu A.M., Braumann C., Lukas C., Uhl W., Künzli B.M. (2021). Lipomatous pancreas facilitates late onset of renal cell carcinoma metastases. Acta Chir. Belg..

[31] Dudani S., de Velasco G., Wells J.C., Gan C.L., Donskov F., Porta C., Fraccon A., Pasini F., Lee J.L., Hansen A. (2021). Evaluation of Clear Cell, Papillary, and Chromophobe Renal Cell Carcinoma Metastasis Sites and Association With Survival. JAMA Netw. Open.

[32] Zhao B., Kimura W., Futakawa N., Muto T., Haida K. (1997). Renal cell carcinoma of the spindle cell type with metastasis to the pancreas: A case report. Jpn. J. Clin. Oncol..

[33] Ayari Y., Ben Rhouma S., Boussaffa H., Chelly B., Hamza K., Sellami A., Jrad M., Nouira Y. (2019). Metachronous isolated locally advanced pancreatic metastasis from chromophobe renal cell carcinoma. Int. J. Surg. Case Rep..

[34] Yano R., Yokota T., Morita M., Amano M., Ochi H., Azemoto N., Mashiba T., Joko K. (2022). A Case of Metastasis from Renal Cell Carcinoma to Ectopic Pancreas Diagnosed after Resection. Intern. Med. Tokyo Jpn..

[35] Piskorz Ł., Mitura K., Olejniczak W., Misiak P., Jablonski S. (2021). Atypical Locations of Renal Cell Carcinoma Metastases to the Pancreas and Duodenum. Res. Rep. Urol..

[36] Alves Ribeiro M., Petersen da Costa Ferreira C., de Lucia Hernani B., Szutan L.A., Galli Mortati M.C., Toledo Bueno Pereira F., Kater F. (2019). Uncommon site of metastasis from renal cell carcinoma: Case report. Int. J. Surg. Case Rep..

[37] Sadhale A., Adike A., Lam-Himlin D. (2018). Metastatic renal cell carcinoma presenting with melena. Clin. Case Rep..

[38] Costa Neves M., Neofytou K., Giakoustidis A., Hazell S., Wotherspoon A., Gore M., Mudan S. (2016). Two cases of gallbladder metastasis from renal cell carcinoma and review of literature. World J. Surg. Oncol..

[39] Chen W.-G., Shan G.-D., Zhu H.-T., Chen L.-H., Xu G.-Q. (2022). Gastric metastasis presenting as submucosa tumors from renal cell carcinoma: A case report. World J. Clin. Cases.

[40] Vo E., Palacio C.H., Omino R., Link R.E., Sada Y., Avo A. (2016). Solitary colon metastasis from renal cell carcinoma nine years after nephrectomy: A case report. Int. J. Surg. Case Rep..

[41] Bruckschen F., Gerharz C.D., Sagir A. (2021). Renal cell carcinoma with unusual metachronous metastasis up to 22 years after nephrectomy: Two case reports. J. Med. Case Rep..

[42] Akhtar S., Usman A., Sultan A., Khalid W., Siddique K. (2022). A Rare Case of Single Gallbladder and Multiple Pancreatic Metastases of Renal Cell Carcinoma. Cureus.

[43] Chung P.H., Srinivasan R., Linehan W.M., Pinto P.A., Bratslavsky G. (2012). Renal cell carcinoma with metastases to the gallbladder: Four cases from the National Cancer Institute (NCI) and review of the literature. Urol. Oncol..

[44] Tapasak B., Mcguirt A. (2022). Metastatic renal cell carcinoma presenting as chronic bleeding from the stomach: A rare case report. J. Surg. Case Rep..

[45] Haidong W., Jianwei W., Guizhong L., Ning L., Feng H., Libo M. (2014). Ampullary tumor caused by metastatic renal cell carcinoma and literature review. Urol. J..

[46] Ignatavicius P., Lizdenis P., Pranys D., Gulbinas A., Pundzius J., Barauskas G. (2018). Long-term Survival of Patient with Ampulla of Vater Metastasis of Renal Cell Carcinoma. Prague Med. Rep..

[47] Cheong D., Rho S.Y., Kim J.H., Kang C.M., Lee W.J. (2018). Laparoscopic pancreaticoduodenectomy for renal cell carcinoma metastasized to ampulla of Vater: A case report and literature review. Ann. Hepato-Biliary-Pancreat. Surg..

[48] Zygulska A.L., Wójcik A., Richter P., Krzesiwo K. (2012). Renal carcinoma metachronous metastases to the gall-bladder and pancreas--case report. Pol. Przegl. Chir..

[49] Lu R., Ying Y., Zhu Z., Wan H., Li G., Shu X., Liao W. (2021). A case report of the pancreatic and periampullary metastases of renal cell carcinoma, 17 years after surgery. Transl. Cancer Res..

[50] Jideh B., Chen H., Weltman M., Chan C.H.Y. (2016). Metastatic periampullary clear cell renal carcinoma. Gastrointest. Endosc..

[51] Hashimoto M., Miura Y., Matsuda M., Watanabe G. (2001). Concomitant duodenal and pancreatic metastases from renal cell carcinoma: Report of a case. Surg. Today.

[52] Ricci V., Carbone S.F., Testi W., Malatesti R., Lo Gatto M., Dell’Avanzato R., Ginanneschi C., Volterrani L. (2008). Single gallbladder and multiple pancreatic metastases from renal cell carcinoma sixteen years after nephrectomy. Chir. Ital..

[53] Momose H., Suzuki Y., Shibahara J., Sakamoto Y. (2021). Metastatic renal cell carcinoma to the pancreas with tumor thrombus in the main pancreatic duct. Jpn. J. Clin. Oncol..

[54] Palmowski M., Hacke N., Satzl S., Klauss M., Wente M.N., Neukamm M., Kleeff J., Hallscheidt P. (2008). Metastasis to the pancreas: Characterization by morphology and contrast enhancement features on CT and MRI. Pancreatol. Off. J. Int. Assoc. Pancreatol. IAP Al.

[55] Majhail N.S., Urbain J.-L., Albani J.M., Kanvinde M.H., Rice T.W., Novick A.C., Mekhail T.M., Olencki T.E., Elson P., Bukowski R.M. (2003). F-18 fluorodeoxyglucose positron emission tomography in the evaluation of distant metastases from renal cell carcinoma. J. Clin. Oncol. Off. J. Am. Soc. Clin. Oncol..

[56] Elahmadawy M.A., Elazab M.S.S., Ahmed S., Salama M. (2018). Diagnostic value of F-18 FDG PET/CT for local and distant disease relapse surveillance in surgically treated RCC patients: Can it aid in establishing consensus follow up strategy?. Nucl. Med. Rev. Cent. East. Eur..

[57] Okasha H.H., Al-Gemeie E.H., Mahdy R.E. (2013). Solitary pancreatic metastasis from renal cell carcinoma 6 years after nephrectomy. Endosc. Ultrasound.

[58] Seicean A., Mosteanu O., Seicean R. (2017). Maximizing the endosonography: The role of contrast harmonics, elastography and confocal endomicroscopy. World J. Gastroenterol..

[59] Iglesias-Garcia J., Lindkvist B., Lariño-Noia J., Abdulkader-Nallib I., Dominguez-Muñoz J.E. (2017). Differential diagnosis of solid pancreatic masses: Contrast-enhanced harmonic (CEH-EUS), quantitative-elastography (QE-EUS), or both?. United Eur. Gastroenterol. J..

[60] Ignee A., Jenssen C., Arcidiacono P.G., Hocke M., Möller K., Saftoiu A., Will U., Fusaroli P., Iglesias-Garcia J., Ponnudurai R. (2018). Endoscopic ultrasound elastography of small solid pancreatic lesions: A multicenter study. Endoscopy.

[61] Eloubeidi M.A., Jhala D., Chhieng D.C., Jhala N., Eltoum I., Wilcox C.M. (2002). Multiple late asymptomatic pancreatic metastases from renal cell carcinoma: Diagnosis by endoscopic ultrasound-guided fine needle aspiration biopsy with immunocytochemical correlation. Dig. Dis. Sci..

[62] Kawakami H., Kuwatani M., Yamato H., Shinada K., Hirano S., Kondo S., Yonemori A., Matsuno Y., Asaka M. (2008). Pancreatic metastasis from renal cell carcinoma with intraportal tumor thrombus. Intern. Med. Tokyo Jpn..

[63] Pannala R., Hallberg-Wallace K.M., Smith A.L., Nassar A., Zhang J., Zarka M., Reynolds J.P., Chen L. (2016). Endoscopic ultrasound-guided fine needle aspiration cytology of metastatic renal cell carcinoma to the pancreas: A multi-center experience. CytoJournal.

[64] Rupert K., Kural T., Skalický T., Zeithaml J., Hess O., Třeška V. (2020). Clear cell renal carcinoma metastases to the pancreas. Rozhl. V Chir..

[65] Krishna S.G., Bhattacharya A., Ross W.A., Ladha H., Porter K., Bhutani M.S., Lee J.H. (2015). Pretest prediction and diagnosis of metastatic lesions to the pancreas by endoscopic ultrasound-guided fine needle aspiration. J. Gastroenterol. Hepatol..

[66] Béchade D., Palazzo L., Fabre M., Algayres J.-P. (2003). EUS-guided FNA of pancreatic metastasis from renal cell carcinoma. Gastrointest. Endosc..

[67] Liang X.-K., Li L.-J., He Y.-M., Xu Z.-F. (2022). Misdiagnosis of pancreatic metastasis from renal cell carcinoma: A case report. World J. Clin. Cases.

[68] De Luca L., Tommasoni S., Mangiavillano B., Repici A. (2021). Metastatic renal cell carcinoma of the pancreas mimicking neuroendocrine tumor diagnosed by endoscopic ultrasound-guided needle biopsy. Clin. Case Rep..

[69] Bhutani M.S., Singh B.S., Cazacu I.M., Saftoiu A. (2019). Differentiating intrapancreatic accessory spleen from a pancreatic neuroendocrine tumor or metastasis by the “bridge sign”. Endosc. Ultrasound.

[70] Kanthan G.L., Schembri G.P., Samra J., Roach P., Hsiao E. (2016). Metastatic Renal Cell Carcinoma in the Thyroid Gland and Pancreas Showing Uptake on 68Ga DOTATATE PET/CT Scan. Clin. Nucl. Med..

[71] Kang T.W., Kim S.H., Lee J., Kim A.Y., Jang K.M., Choi D., Kim M.J. (2015). Differentiation between pancreatic metastases from renal cell carcinoma and hypervascular neuroendocrine tumour: Use of relative percentage washout value and its clinical implication. Eur. J. Radiol..

[72] Minezaki S., Misawa T., Tsukayama H., Shibuya M., Wada K., Sano K., Mochizuki M., Sasajima Y., Kondo H. (2022). Tumor-to-tumor metastasis: An extremely rare combination with renal cell carcinoma as the donor and a pancreatic neuroendocrine tumor as the recipient. Surg. Case Rep..

[73] Bednarek-Rajewska K., Zalewski P., Bręborowicz D., Woźniak A. (2017). Renal cell carcinoma metastasizing to pancreatic neuroendocrine neoplasm—The second case described in the world literature. Pol. J. Pathol. Off. J. Pol. Soc. Pathol..

[74] Shah L., Tiesi G., Bamboat Z., McCain D., Siegel A., Mannion C. (2015). Tumor-to-tumor metastasis: Report of two cases of renal cell carcinoma metastasizing to microcystic serous cystadenoma of the pancreas. Int. J. Surg. Pathol..

[75] Haeberle L., Busch M., Kirchner J., Fluegen G., Antoch G., Knoefel W.T., Esposito I. (2021). Pancreatic ductal adenocarcinoma concomitant with pancreatic metastases of clear-cell renal cell carcinoma: A case report. J. Med. Case Rep..

[76] Mahfoud T., Tanz R., Khmamouche M.R., Allaoui M., Belbaraka R., Khouchani M., Ichou M. (2017). Synchronous Primary Renal Cell Carcinoma and Pancreatic Ductal Adenocarcinoma: Case Report and Literature Review. Case Rep. Oncol..

[77] Gnemmi V., Leroy X., Triboulet J.-P., Pruvot F.-R., Villers A., Leteurtre E., Buob D. (2013). Pancreatic metastases of renal clear cell carcinoma: A clinicopathological study of 11 cases with special emphasis on the usefulness of PAX2 and mesothelin for the distinction from primary ductal adenocarcinoma of the pancreas. Anal. Quant. Cytopathol. Histopathol..

[78] Sharma S.G., Gokden M., McKenney J.K., Phan D.C., Cox R.M., Kelly T., Gokden N. (2010). The utility of PAX-2 and renal cell carcinoma marker immunohistochemistry in distinguishing papillary renal cell carcinoma from nonrenal cell neoplasms with papillary features. Appl. Immunohistochem. Mol. Morphol..

[79] Kang Y.-N. (2022). Primary Clear Cell Carcinoma of the Pancreas: A Rare Case Report. Diagnostics.

[80] Yamada Y., Sakai A., Abe S., Gonda M., Kobayashi T., Masuda A., Shiomi H., Shirakawa S., Toyama H., Hyodo T. (2021). Pancreatic metastasis of renal cell carcinoma filling into the duct of Santorini. Clin. J. Gastroenterol..

[81] Shin T.J., Song C., Jeong C.W., Kwak C., Seo S., Kang M., Chung J., Hong S.-H., Hwang E.C., Park J.Y. (2021). Metastatic renal cell carcinoma to the pancreas: Clinical features and treatment outcome. J. Surg. Oncol..

[82] Chin W., Cao L., Liu X., Ye Y., Liu Y., Yu J., Zheng S. (2020). Metastatic renal cell carcinoma to the pancreas and subcutaneous tissue 10 years after radical nephrectomy: A case report. J. Med. Case Rep..

[83] Wu C., Zhou Z., Ye X., Hu W. (2016). Synchronous renal cell carcinoma metastasis to the contralateral adrenal gland and pancreas: A case report with 7-year follow-up subsequent to surgical therapy. Oncol. Lett..

[84] Al Abdrabalnabi A.A., AlQattan A.S., Algarni S., Mashhour M., Al-Qahtani M. (2019). Metastatic renal cell carcinoma to the pancreas, thyroid, & subcutaneous tissue 13 years after Radical nephrectomy: A case report. Int. J. Surg. Case Rep..

[85] Sperti C., Moletta L., Patanè G. (2014). Metastatic tumors to the pancreas: The role of surgery. World J. Gastrointest. Oncol..

[86] Cardoso D., Rosales A., Thiel D.D., Asbun H., Stauffer J.A. (2022). Pancreatic metastasectomy of renal cell carcinoma: A single institution experience. Can. J. Urol..

[87] Blanco-Fernández G., Fondevila-Campo C., Sanjuanbenito A., Fabregat-Prous J., Secanella-Medayo L., Rotellar-Sastre F., Pardo-Sánchez F., Prieto-Calvo M., Marín-Ortega H., Sánchez-Cabús S. (2022). Pancreatic metastases from renal cell carcinoma. Postoperative outcome after surgical treatment in a Spanish multicenter study (PANMEKID). Eur. J. Surg. Oncol. J. Eur. Soc. Surg. Oncol. Br. Assoc. Surg. Oncol..

[88] Malleo G., Salvia R., Maggino L., Marchegiani G., D’Angelica M., DeMatteo R., Kingham P., Pulvirenti A., Sereni E., Jarnagin W.R. (2021). Long-term Outcomes After Surgical Resection of Pancreatic Metastases from Renal Clear-Cell Carcinoma. Ann. Surg. Oncol..

[89] Glinka J., Sanchez Claria R., Ardiles V., de Santibañes E., Pekolj J., de Santibañes M., Mazza O. (2019). The pancreas as a target of metastasis from renal cell carcinoma: Results of surgical treatment in a single institution. Ann. Hepato-Biliary-Pancreat. Surg..

[90] Faure J.P., Tuech J.J., Richer J.P., Pessaux P., Arnaud J.P., Carretier M. (2001). Pancreatic metastasis of renal cell carcinoma: Presentation, treatment and survival. J. Urol..

[91] Yazbek T., Gayet B. (2012). The place of enucleation and enucleo-resection in the treatment of pancreatic metastasis of renal cell carcinoma. JOP J. Pancreas.

[92] Niess H., Conrad C., Kleespies A., Haas F., Bao Q., Jauch K.-W., Graeb C., Bruns C.J. (2013). Surgery for metastasis to the pancreas: Is it safe and effective?. J. Surg. Oncol..

[93] Sohn T.A., Yeo C.J., Cameron J.L., Nakeeb A., Lillemoe K.D. (2001). Renal cell carcinoma metastatic to the pancreas: Results of surgical management. J. Gastrointest. Surg. Off. J. Soc. Surg. Aliment. Tract.

[94] Fikatas P., Klein F., Andreou A., Schmuck R.B., Pratschke J., Bahra M. (2016). Long-term Survival After Surgical Treatment of Renal Cell Carcinoma Metastasis Within the Pancreas. Anticancer Res..

[95] Tosoian J.J., Cameron J.L., Allaf M.E., Hruban R.H., Nahime C.B., Pawlik T.M., Pierorazio P.M., Reddy S., Wolfgang C.L. (2014). Resection of isolated renal cell carcinoma metastases of the pancreas: Outcomes from the Johns Hopkins Hospital. J. Gastrointest. Surg. Off. J. Soc. Surg. Aliment. Tract.

[96] Yamaguchi H., Kimura Y., Nagayama M., Imamura M., Tanaka S., Yoshida M., Yoshida E., Fujino H., Machiki T., Miyanishi K. (2019). Central pancreatectomy in portal annular pancreas for metastatic renal cell carcinoma: A case report. World J. Surg. Oncol..

[97] Volk A., Kersting S., Konopke R., Dobrowolski F., Franzen S., Ockert D., Grutzmann R., Saeger H.D., Bergert H. (2009). Surgical therapy of intrapancreatic metastasis from renal cell carcinoma. Pancreatol. Off. J. Int. Assoc. Pancreatol. IAP Al.

[98] Zerbi A., Ortolano E., Balzano G., Borri A., Beneduce A.A., Di Carlo V. (2008). Pancreatic metastasis from renal cell carcinoma: Which patients benefit from surgical resection?. Ann. Surg. Oncol..

[99] Macrì A., Fleres F., Putortì A., Lentini M., Ascenti G., Mastrojeni C. (2014). Relapsed metachronous pancreatic metastasis from renal cell carcinoma (RCC): Report of a case and review of literature. Ann. Ital. Chir..

[100] Sperti C., Pozza G., Brazzale A.R., Buratin A., Moletta L., Beltrame V., Valmasoni M. (2016). Metastatic tumors to the pancreas: A systematic review and meta-analysis. Minerva Chir..

[101] Madkhali A.A., Shin S.-H., Song K.B., Lee J.H., Hwang D.W., Park K.M., Lee Y.J., Kim S.C. (2018). Pancreatectomy for a secondary metastasis to the pancreas: A single-institution experience. Medicine.

[102] Santoni M., Conti A., Partelli S., Porta C., Sternberg C.N., Procopio G., Bracarda S., Basso U., De Giorgi U., Derosa L. (2015). Surgical resection does not improve survival in patients with renal metastases to the pancreas in the era of tyrosine kinase inhibitors. Ann. Surg. Oncol..

[103] Zhang Z.Y., Li X.Y., Bai C.M., Zhou Y., Wu X., Yang A.M., Hua S.R. (2020). The clinicopathologic features and prognostic analysis of pancreatic metastasis from clear cell renal cell carcinoma. Zhonghua Zhong Liu Za Zhi.

[104] Chatzizacharias N.A., Rosich-Medina A., Dajani K., Harper S., Huguet E., Liau S.S., Praseedom R.K., Jah A. (2017). Surgical management of hepato-pancreatic metastasis from renal cell carcinoma. World J. Gastrointest. Oncol..

[105] Itamoto S., Abe T., Oshita A., Hanada K., Nakahara M., Noriyuki T. (2022). Repeat pancreatic resection for metachronous pancreatic metastasis from renal cell carcinoma: A case report. Int. J. Surg. Case Rep..

[106] Hata T., Sakata N., Aoki T., Yoshida H., Kanno A., Fujishima F., Motoi F., Masamune A., Shimosegawa T., Unno M. (2013). Repeated pancreatectomy for metachronous duodenal and pancreatic metastases of renal cell carcinoma. Case Rep. Gastroenterol..

[107] Carrafiello G., Laganà D., Recaldini C., Dionigi G., Boni L., Bacuzzi A., Fugazzola C. (2008). Radiofrequency ablation of a pancreatic metastasis from renal cell carcinoma: Case report. Surg. Laparosc. Endosc. Percutan. Tech..

[108] Karaisz F.G., Elkelany O.O., Davies B., Lozanski G., Krishna S.G. (2023). A Review on Endoscopic Ultrasound-Guided Radiofrequency Ablation (EUS-RFA) of Pancreatic Lesions. Diagnostics.

[109] Barthet M., Giovannini M., Gasmi M., Lesavre N., Boustière C., Napoleon B., LaQuiere A., Koch S., Vanbiervliet G., Gonzalez J.-M. (2021). Long-term outcome after EUS-guided radiofrequency ablation: Prospective results in pancreatic neuroendocrine tumors and pancreatic cystic neoplasms. Endosc. Int. Open.

[110] Dhaliwal A., Kolli S., Dhindsa B.S., Choa J., Mashiana H.S., Ramai D., Chandan S., Bhogal N., Sayles H., Bhat I. (2020). Efficacy of EUS-RFA in pancreatic tumors: Is it ready for prime time? A systematic review and meta-analysis. Endosc. Int. Open.

[111] Crinò S.F., D’Onofrio M., Bernardoni L., Frulloni L., Iannelli M., Malleo G., Paiella S., Larghi A., Gabbrielli A. (2018). EUS-guided Radiofrequency Ablation (EUS-RFA) of Solid Pancreatic Neoplasm Using an 18-gauge Needle Electrode: Feasibility, Safety, and Technical Success. J. Gastrointest. Liver Dis..

[112] Chanez B., Caillol F., Ratone J.-P., Pesenti C., Rochigneux P., Pignot G., Thomassin J., Brunelle S., Walz J., Salem N. (2021). Endoscopic Ultrasound-Guided Radiofrequency Ablation as an Future Alternative to Pancreatectomy for Pancreatic Metastases from Renal Cell Carcinoma: A Prospective Study. Cancers.

[113] Cao H., Sun Z., Wu J., Hao C., Wang W. (2022). Metastatic Clear Cell Renal Cell Carcinoma to Pancreas and Distant Organs 24 Years After Radical Nephrectomy: A Case Report and Literature Review. Front. Surg..

[114] Medioni J., Choueiri T.K., Zinzindohoué F., Cho D., Fournier L., Oudard S. (2009). Response of renal cell carcinoma pancreatic metastasis to sunitinib treatment: A retrospective analysis. J. Urol..

[115] Nogueira M., Dias S.C., Silva A.C., Pinto J., Machado J. (2018). Solitary pancreatic renal cell carcinoma metastasis. Autopsy Case Rep..

[116] Jiang W., Shi H., Zhang L., Zhang J., Bi X., Wang D., Wen L., Li C., Ma J., Shou J. (2020). Responses to Targeted Therapy among Organs Affected by Metastasis in Patients with Renal Cell Carcinoma are Organ-Specific. Urol. J..

[117] Negishi T., Furubayashi N., Nakagawa T., Nishiyama N., Kitamura H., Hori Y., Kuroiwa K., Son Y., Seki N., Tomoda T. (2021). Site-specific Response to Nivolumab in Renal Cell Carcinoma. Anticancer Res..

