# Supplementary material for: Metastatic Renal Cell Carcinoma to Pancreas: Case Series and Review of the Literature
Source: Diagnostics (Basel). 2023 Apr 7;13(8):1368. doi: 10.3390/diagnostics13081368 (PMC10137405; doi:10.3390/diagnostics13081368)
Supplement: Supplementary file 1 [file diagnostics-13-01368-s001.zip › diagnostics-2254516-supplementary.pdf]

Supplementary Table S1: Summary of clinical and diagnostic features of pancreatic metastatic lesions.

Author, year	Nr. of pts. with PM	Time from RCC diagnosis (median)	Clinical features	Imaging	No. of metastatic lesions	Diagnostic method
Li J et al., 2019 [5]	1	19 y	palpable mass	CT	1	surgery
Wente MN et al., 2005 [15]	15	86 mo. (range 0-258)<br><br>1 synchronous	most asymptomatic	N/A	10 solitary<br><br>5 multiple	surgery
Benhaim R et al., 2015 [16]	20	130 ± 59 mo. (range 24-240)	55% asymptomatic<br><br>45 % symptomatic: asthenia (44%), GI bleeding (22%), abdominal pain (22%), pancreatic insufficiency (12%)	CT, endoscopy	13 solitary<br><br>7 multiple	surgery in all pts.<br><br>pre-surgery-FNB in 40%
Moletta L et al., 2014 [17]	13	6 y (range 1-22)	jaundice and abdominal pain in 3 pts.	US, CT<br><br>4 underwent Octreoscan	4 solitary<br><br>multiple (up to 6)	urgery in 9 pts. (EUS not available)
Thompson LD et al., 2000 [18]	21	8.4 y (range 0.5-27)	abdominal pain in 7 pts.<br><br>early satiety, weight loss, diarrhea, GI bleeding symptoms associated with pancreatic insufficiency in 9 pts.<br><br>5 asymptomatic	abdominal US, CT, MRI, ERCP (in pts. with bile duct obstruction)	11 solitary<br><br>9 multiple<br><br>1 unknown	surgery
Gilbert CM et al., 2011 [19]	12	8.8 y (range 2-19)	N/A	N/A	8 solitary<br><br>4 multiple	FNA + surgery
Abdul-Ghafar J et al., 2021 [20]	2	11 and 14 y	abdominal pain in 1 pt.	CT	solitary	CT guided core needle biopsy + surgery
Nakamura H et al., 2021 [21]	1	36 y	N/A	CT	solitary	EUS-FNA
Matsui S et al., 2021 [22]	1	17 y	melena with signs of shock	endoscopy, CT + PET-CT	solitary	surgery
Yamawaki M et al., 2022 [23]	1	22 y	right hypochondrial pain, fever, jaundice	CT + MRI<br><br>ERCP	multiple	EUS-FNA
Gajendra S et al., 2015 [24]	1	3 y	acute abdomen	CT	solitary	surgery
Karakatsanis A et al., 2013 [25]	1	3 y	jaundice	US, CT<br><br>ERCP	Single ampullary	ampullectomy

Schauer M et al., 2008 [26]	10	110 mo. (range 5-277)	jaundice + abdominal pain in 3 pts.<br><br>GI bleeding in 3 pts.<br><br>4 asymptomatic	CT, MRI, US/EUS	3 solitary<br><br>7 multiple	surgery
Ghavamian R et al., 2000 [29]	23	116 mo. (range 1-295)	NOD in 3 pts., GI bleeding in 1 pt.	CT	12 solitary<br><br>11 multiple	biopsy using CT (10 pts.), US (2 pts.), ERCP (2 pts.), or surgery (9 pts.).
Zhao B et al., 1997 [32]	1	synchronous	abdominal pain, jaundice	CT	multiple	lymph node biopsy
Ayari Y et al., 2019 [33]	1	2 y	asymptomatic	CT	solitary	CT guided core needle biopsy
Piskorz Ł et al., 2021 [35]	1	7 y	acute pancreatitis	CT	multiple	surgery
Alves Ribeiro M et al., 2019 [36]	1	9 y	asymptomatic	CT + MRI	solitary	EUS-FNA/FNB
Sadhale A et al., 2018 [37]	1	13 y	melena	CT	multiple	N/A
Bruckschen F et al., 2021 [41]	1	21 y	GI bleeding	endoscopy, CT	solitary	endoscopic biopsy from duodenal bulb
Akhtar S et al., 2022 [42]	1	synchronous	hematuria	CT	multiple	EUS-FNA
Haidong W et al., 2014 [45]	1	synchronous	jaundice	CT	Single ampullary	surgery
Ignatavicius P et al., 2018 [46]	1	8 mo.	upper abdominal pain, jaundice, general weakness	CT	Single ampullary	endoscopic biopsy
Cheong D et al., 2018 [47]	1	12 y	jaundice	MRI, PET-CT	Single ampullary and pancreatic	EUS-FNA
Zygulska AL et al., 2012 [48]	1	47 mo.	asymptomatic	CT	solitary	surgery
Lu R et al., 2021 [49]	1	17 y	jaundice, pruritus, hematochezia	US, CT	Single ampullary and pancreatic	endoscopic biopsy
Hashimoto M et al., 2001 [51]	1	11 y	GI bleeding	CT, MRI	solitary	surgery
Ricci V et al., 2008 [52]	1	16 y	GI bleeding	CT	multiple	surgery
Momose H et al., 2020 [53]	1	4 y	asymptomatic	CT	solitary	surgery
Okasha HH et al., 2013 [57]	1	6 y	abdominal pain	US, CT	solitary	EUS-FNA
Pannala R et al., 2016 [63]	33	6.8 y (range 1-14)	N/A	CT, MRI, EUS	8 solitary<br><br>25 multiple	EUS-FNA
Rupert K et al., 2020 [64]	12	8.6 y	N/A	CT, EUS	N/A	EUS-FNA
Béchade D et al., 2003 [66]	11	92 mo. (range 0-228)	jaundice in 4 pts. and abdominal pain in 3 pts.	US, CT	5 solitary<br><br>6 multiple	EUS-FNA

Liang XK et al., 2022 [67]	1	19 y	asymptomatic	CT	solitary	EUS-FNB
Yamada Y et al., 2021 [80]	1	18 y	abdominal pain	CT	solitary	surgery
Shin TJ et al., 2021 [81]	300	104 synchronous<br><br>196 metachronous – median 82 mo. (range 31–141)	50% asymptomatic<br><br>31.3 % symptomatic<br><br>18.7% unknown	CT	N/A	metastasectomy in 198 pts.
Chin W et al., 2020 [82]	1	10 y	asymptomatic	CT	solitary	surgery
Wu C et al., 2016[83]	1	synchronous	asymptomatic	CT, MRI	solitary	surgery
Al Abdrabalnabi AA et al., 2019 [84]	1	6 y	asymptomatic	CT	multiple	surgery
Blanco-Fernández G et al., 2022 [87]	116	87.3 mo.<br><br>2 synchronous	86.2% asymptomatic<br><br>13.8% symptomatic – abdominal pain, jaundice, weight loss	CT, MRI, EUS	95 solitary<br><br>21 multiple	surgery
Malleo G et al., 2021 [88]	69	109 mo. (range 0-294)<br><br>9 synchronous	N/A	N/A	59 solitary<br><br>10 multiple	surgery
Glinka J et al., 2019 [89]	8	9.2 y (range 1–24.8)	asymptomatic	CT	7 solitary<br><br>1 multiple	surgery
Niess H et al., 2013 [92]	16	5.3 y (range 0-24)	N/A	N/A	12 solitary<br><br>4 multiple	surgery
Tosoian JJ et al., 2014 [95]	42	1.2 y (range 0–28)<br><br>4 synchronous	54.8% asymptomatic<br><br>45.2% symptomatic – weight loss, abdominal pain, GI bleeding, jaundice	N/A	24 solitary<br><br>18 multiple	surgery
Volk A et al., 2009 [97]	14	94 mo. (range 32–158)	10 asymptomatic<br><br>jaundice in 3 pts.<br><br>back pain in 1 pt.	US, CT	10 solitary<br><br>4 multiple	surgery
Zerbi A et al., 2008 [98]	36	8 y (range 1–23)	77.8% asymptomatic<br><br>weight loss and malaise in 8.3%, jaundice in 5.6%, anemia in 5.6%, and abdominal pain in 2.8%	CT	26 solitary<br><br>10 multiple	surgery in 23 pts.<br><br>surgical biopsy in 1 pt.<br><br>percutaneous FNA in 5 pts.<br><br>EUS-FNA in 7 pts.

Santoni M et al., 2015 [102]	103	9.6 y (range 0–24)<br><br>3 synchronous	72 asymptomatic<br><br>26 symptomatic: pain (10%), asthenia (7%), other symptoms (4%)	CT, MRI	for pts. with ancreas as only etastatic site (n = 56):<br><br>36 solitary<br><br>8 multiple	N/A
Zhang ZY et al., 2020 [103]	18	156 mo.<br><br>3 synchronous	abdominal pain, jaundice, GI bleeding, nausea, weakness, weight loss	N/A	7 solitary<br><br>11 multiple	N/A
Chatzizacharias et al., 2017 [104]	7	80 mo. (range 10-292)<br><br>1 synchronous	N/A	CT, PET-CT, MRI	N/A	EUS-FNA
Cao H et al., 2022 [113]	1	24 y	epigastric pain, abdominal distention, nausea, vomiting	CT, PET-CT	solitary	Metastasectomy
Medioni J et al., 2009 [114]	15	7 y (range 0 - 12)	abdominal pain in 1 pt.	CT	9 solitary<br><br>6 multiple	N/A
Nogueira M et al., 2018 [115]	1	16 y	abdominal pain	CT, MRI	solitary	Surgery

RCC = renal cell carcinoma; pt. = patients; pts. = patients; y = year(s); mo. = month(s); N/A = not applicable/not available; CT = computer tomography; PET-CT = positron emission tomography; MRI = magnetic resonance imaging; US = ultrasound; ERCP = endoscopic retrograde cholangiopancreatography; EUS = endoscopic ultrasound; FNA = fine needle aspiration; FNB = fine needle biopsy; GI = gastrointestinal; NOD = new-onset diabetes mellitus.
